# Exploratory Analysis of Age-Related Trends in Biostimulatory Response to Combined Calcium Hydroxylapatite-Carboxymethylcellulose and Microfocused Ultrasound With Visualization Treatments

**DOI:** 10.1093/asjof/ojaf023

**Published:** 2025-04-16

**Authors:** Amanda Doyle, Iris Looi, Paul Chu, Keith A Martinez, Alec D McCarthy

## Abstract

**Background:**

Biostimulators and energy-based devices are increasingly used in combination to address visible signs of aging. Previous research demonstrated that pairing microfocused ultrasound with visualization (MFU-V) and hyperdiluted calcium hydroxylapatite-carboxymethylcellulose (CaHA-CMC) enhances dermal elastin synthesis and improves aesthetic outcomes up to 120 days posttreatment. However, the impact of patient age on the histological response to these treatments remains unclear.

**Objectives:**

To explore whether age influences the biostimulatory response to combined hyperdiluted CaHA-CMC and MFU-V treatments in healthy adults aged 39 to 62 years.

**Methods:**

This secondary analysis leveraged data from a 12-patient, IRB-approved clinical study in which patients received 2 combination treatment protocols: Group A (MFU-V followed by hyperdiluted CaHA-CMC) and Group B (hyperdiluted CaHA-CMC followed by MFU-V). Biopsies obtained before and 120 days after completion of treatments were stained for elastin and quantitatively assessed. Linear regression analyses assessed correlations between age and changes in elastin staining intensity and area coverage.

**Results:**

No significant correlation was found between patient age and changes in dermal elastin intensity or area. In subgroup analyses, neither protocol showed a significant age-dependent difference in elastin intensity. Although 1 subgroup (Group B) revealed a marginal correlation between age and elastin area, the pooled data did not support age as a significant predictor of biostimulatory response.

**Conclusions:**

This exploratory analysis suggests that within the studied age range, patient age may not significantly influence the histological response to combined CaHA-CMC and MFU-V treatments. Larger, statistically powered studies are needed to validate these findings and further investigate age-related effects.

**Level of Evidence: 5 (Therapeutic):**

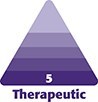

Combination biostimulatory procedures, namely the pairing of injectable regenerative biostimulators and the use of energy-based devices (EBDs), are common in clinical practice.^1^ Our recent comparative histopathologic study sought to determine the histological and aesthetic effects of treatment protocols combining calcium hydroxylapatite-carboxymethylcellulose (CaHA-CMC; Radiesse, Merz North America, Inc., Raleigh, NC) and microfocused ultrasound with visualization (MFU-V; Ultherapy, Ulthera, Inc., Raleigh, NC) administered in 2 different timing protocols.^2^ CaHA-CMC is a regenerative biostimulator consisting of uniform CaHA microspheres that promote collagen and elastin fibrillogenesis through fibroblast mechanotransduction.^3-5^ MFU-V is a noninvasive, heat-based therapy that deposits thermal coagulation points that result in collagen and elastin fibrillogenesis via an acute inflammatory response followed by extracellular matrix (ECM) remodeling.^1,6,7^

In our previous study, 12 healthy female patients were randomized into 2 groups. Group A (*n* = 6) first received a treatment of MFU-V followed by a treatment of hyperdiluted CaHA-CMC, whereas Group B (*n* = 6) first received a treatment of hyperdiluted CaHA-CMC followed by a treatment of MFU-V. Each patient underwent photographic and histological analysis (Verhoff Van Gieson staining for elastin) with biopsies taken before treatment and 120 days after the second treatment. The results from this study showed significant qualitative improvements in patient appearance and quantitative improvements in dermal elastin staining intensity and area coverage for both groups. Group A experienced a significantly greater improvement in elastin synthesis, which correlated with an overall improved aesthetic outcome based on patient and investigator Global Aesthetic Improvement Scales (sGAIS and iGAIS, respectively), and FACE-Q measurement compared with Group B.

In addition to demonstrating an optimal cadence between biostimulators and EBD treatment, our study allowed us to assess whether age significantly affected responses to biostimulation. To our knowledge, no study has explored the effect of age on histological responses to biostimulatory aesthetic treatments. Although it is known that fibroblast function, collagen and elastin content, and ECM regeneration decrease with age, it is unknown whether this significantly affects ECM regeneration posttreatment.^8-10^ Chronological aging and its associated fibroblast collapse thus raise the question of whether there is an optimal age range for patients undergoing biostimulatory treatments and whether or not age significantly affects response to such treatments. Using ECM remodeling data from our primary publication and the enrolled patient ages, we conducted both pooled and subgroup regression analysis to determine to what extent, if any, age affected patients’ response to biostimulation.

## METHODS

This report is a secondary analysis of data from Doyle et al's IRB-approved, patient-consented study. Briefly, 12 patients were randomized into 2 groups after enrollment in the study. All patients completed the study. Group A (*n* = 6) received a treatment of MFU-V at multiple depths first, followed by a treatment of hyperdiluted (1:2) CaHA, whereas Group B (*n* = 6) received a treatment of hyperdiluted (1:2) CaHA first, followed by a treatment of MFU-V at multiple depths. Treatments were spaced 42 ± 7 days apart. Biopsies before and 120 days after treatment were taken for each patient and quantitatively evaluated using ImageJ based on a simple protocol proposed by Chen et al adapted for elastin.^11,12^ The changes in elastin staining area and intensity were paired with that patient's age, and a series of pooled and subgroup simple linear regression analyses were carried out. Both Pearson's *r* and *R*^2^ were calculated to provide insight into the goodness of fit of the regression as well as the direct linear relationship between each variable. In addition, BMI, which was gathered at baseline, Day 90 ± 7, and Day 120 ± 7, and age were compared using 2- and 1-way analysis of variances. Significance values were denoted in pairwise comparisons as follows: ns = *P* > .05, *P < .05, **P < .01, ***P < .001, ****P < .00001. All statistical analysis and graphing were performed using GraphPad Prism version 10.2.3 (GraphPad LLC, San Diego, CA).

## RESULTS

**Figure 1.**
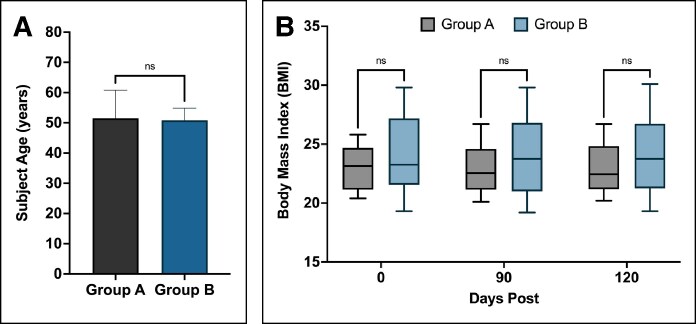
(A) Average patient age in Groups A and B. (B) Average BMIs of patients in Groups A and B throughout the course of the study.

**Figure 2.**
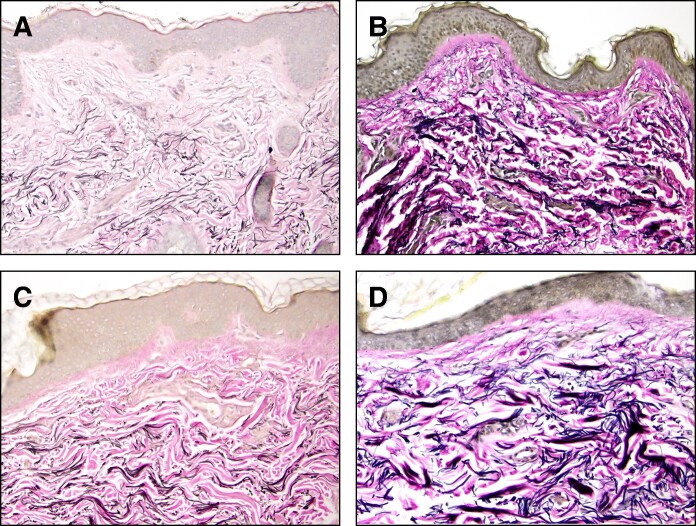
Elastin van Gieson stain highlighting elastic fibers (black) (A) before and (B) 120 days after combined MFU-V/CaHA-CMC treatment in a 39-year-old patient and (C) before and (D) 120 days after combined MFU-V/CaHA-CMC treatment in a 62-year-old patient. CaHA-CMC, calcium hydroxylapatite-carboxymethylcellulose; MFU-V, microfocused ultrasound with visualization.

The average age of the entire 12-patient group was 51.17 ± 6.86 years (range, 39-62 years), whereas the average age of Groups A and B was 51.50 ± 9.33 years (range, 39-62 years) and 50.83 ± 4.021 years (range, 47-58 years), respectively (Figure 1A). The average age of each group did not vary significantly (*P* = .8755), nor did their BMI (*P* = .5360; Figure 1B). BMI was static throughout the study, with Groups A and B having average BMIs of 23.03 ± 2.001 (range, 20.4-25.8) and 24.03 ± 3.64 (range, 19.3-29.8) at baseline and 22.92 ± 2.35 (range, 20.2-26.7) and 24.07 ± 3.68 (range, 19.3-30.1) at Day 120, respectively. The majority of patients treated experienced increases in dermal elastin (92%; 11/12). The proportion of patients with increased dermal elastin was 100% (6/6) in Group A and 83% (5/6) in Group B. Elastin stains of 39- and 62-year-old patients are visualized in Figure 2.

**Figure 3.**
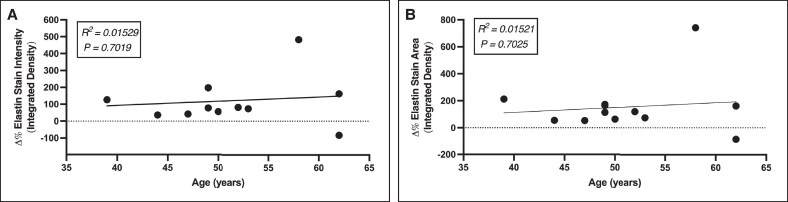
Pooled linear regression analysis illustrating the lack of significant correlation between patient age and (A) change in histological elastin stain intensity (brightness of stain) and (B) change in elastin-positive areas of histological samples. Although 12 data points are present, 2 data points overlap because of similar values and may appear as a single point at this resolution.

**Figure 4.**
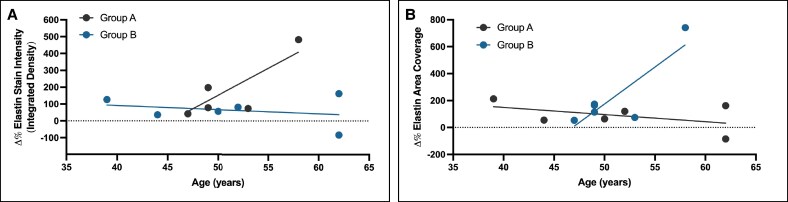
Subgroup linear regression analysis illustrating the relationship between patient age and (A) change in histological elastin stain intensity (brightness of stain) and (B) change in elastin-positive areas of histological samples in Groups A and B. Although 12 data points are present, 2 data points overlap because of similar values and may appear as a single point at this resolution.

When assessing the entire group (*n* = 12), age and change in both dermal elastin staining intensity (*R*^2^ = 0.01529; *P* = .7019) and dermal elastin staining area (*R*^2^ = 0.01521; *P* = .7025) were not significantly correlated (Figure 3A, B). When assessing by subgroup, changes in dermal elastin staining intensity were not significantly correlated with age in either group (Group A: *R*^2^ = 0.6248; *P* = .0613, Group B: *R*^2^ = 0.07257; *P* = .60573; Figure 4A). Subgroup changes in dermal elastin staining area for Group A were not significantly correlated with age (*R*^2^ = 0.2278; *P* = .3384), whereas such changes were marginally significant in Group B (*R*^2^ = 0.723; *P* = .0319; Figure 4B).

## DISCUSSION

Although the authors of other studies have compared histological and aesthetic changes by altering biostimulatory and EBD treatment cadence,^13^ this is the first study where the authors have assessed the role of age on response to the combined biostimulation. Results from this exploratory analysis suggest that age did not correlate significantly with response to biostimulation and support the use of such treatments in a range of patient ages. However, a weak correlation was detected in Group B, which may be explained by the increasingly delayed healing response with increasing age.^14^ Since Group B underwent MFU-V second, they had less time to progress through the entire remodeling phase of thermal healing, which is particularly impaired with increasing age.^15^ Although the age range in our study was sizable (23 years), it is unclear whether patients older than 62 would follow a similar trend. Beyond a certain age, significant fibroblast collapse likely reduces the efficacy of biostimulatory treatments. This threshold varies for each patient based on factors such as diet, protein intake, amino acid availability, and metabolic health. For example, it has been previously demonstrated that vegans responded significantly worse to MFU-V treatments than omnivorous patients.^16^

Overall, this subanalysis revealed a trend suggesting that age was not a significant predictor in ECM-stimulation response to combined CaHA-CMC and MFU-V treatments in patients aged 39 to 62 years.

### Limitations

Several limitations must be acknowledged. First, the small sample size (*n* = 12) limits the statistical power of regression analyses, preventing definitive conclusions. Given the lack of statistical power, the results should be interpreted as observational trends rather than a definitive lack of relationship. Second, the age range of 39 to 62 years excludes younger and older populations, which limits the generalizability of the findings, which is especially relevant when extrapolating to older populations. Despite these limitations, the study's strengths include its novel focus on age-related biostimulatory responses and its quantitative analysis of elastin remodeling and statistical analysis. These findings provide a valuable basis for future, more robust investigations into the effects of age on regenerative aesthetic treatments.

## CONCLUSIONS

This exploratory analysis assessed the impact of age on the biostimulatory response to combined CaHA and MFU-V treatments in healthy patients aged 39 to 62 years. The results demonstrated that age did not significantly affect changes in dermal elastin staining intensity or area, suggesting that patients within this age range may benefit similarly from biostimulatory treatments. Although this study identified a potential trend, the findings remain inconclusive due to limited sample size and insufficient statistical power. Larger, well-powered studies with wider age ranges must be conducted to determine the extent to which this effect is observed.
